# Phenotype- and phase-directed immune interpretation in recurrent implantation failure and pregnancy loss: an integrative framework

**DOI:** 10.3389/frph.2026.1780022

**Published:** 2026-04-15

**Authors:** Ahmed Elgheriany

**Affiliations:** Consultant Gynaecologist and Fertility Specialist, Newlife Fertility Clinic, Epsom, United Kingdom

**Keywords:** cytokine balance, immune phenotyping, implantation window, natural killer cells, recurrent implantation failure, recurrent pregnancy loss, reproductive immunology

## Abstract

Recurrent implantation failure (RIF) and recurrent pregnancy loss (RPL) remain major challenges in reproductive medicine. Although immune mechanisms are integral to implantation, clinical translation has been limited by indiscriminate use of immune-directed therapies in unselected populations, mistimed immune assessment outside the implantation window, and insufficient distinction between immune-mediated failure and non-immune causes, contributing to inconsistent or null outcomes in randomised trials of empiric immunotherapy. To address these limitations, this review introduces the Elgheriany Reproductive Immunology Framework (ERIF) as a conceptual interpretive model integrating prerequisite exclusion, synchronised immune profiling within the implantation window, and cytokine-informed consideration of immunomodulation initiation and withdrawal. The framework emphasises interpretation of immune findings rather than presumption of immune causality and conditions intervention on demonstrable dysfunction. Within this context, a subset of patients may exhibit failure of the normal transition from early inflammatory activation to immune tolerance, reflected by persistent Th1-skewed cytokine activity and, in selected phenotypes, altered natural killer (NK) cell cytotoxicity. Peripheral and uterine immune compartments are interpreted jointly, recognising their functional divergence and temporal specificity during implantation. ERIF is intended to support immune interpretation and the design of phenotype-stratified clinical trials rather than to function as a prescriptive therapeutic algorithm, providing a basis for future validation in recurrent reproductive failure.

## 1 Introduction

Implantation is no longer viewed as a static tolerant state but as a dynamic immune choreography. A transient inflammatory burst is required for blastocyst apposition and adhesion, after which rapid induction of tolerance and angiogenesis allows invasion, vascular remodelling, and early placentation (1, 2). When this transition fails, whether due to persistent Th1 dominance or inadequate tolerance induction, clinical sequelae manifest as recurrent implantation failure (RIF) or recurrent pregnancy loss (RPL) (3, 4).

Despite decades of mechanistic insight, clinical translation in reproductive immunology has remained fragmented. Three recurring obstacles explain this impasse: heterogeneity of assays, including differences in platforms, gating strategies, and thresholds; mistimed sampling outside the narrow implantation window; and therapeutic empiricism, whereby immunosuppressive agents such as corticosteroids, intralipids, or intravenous immunoglobulin (IVIG) are applied to immunologically unselected patients (5, 6). Unsurprisingly, randomised trials conducted in such unstratified populations have largely yielded null results.

The unmet clinical need, therefore, is not broader immunosuppression but precision selection: identifying the minority of patients with genuine immune dysregulation, defining their phenotype, and intervening only to the degree and duration required. This approach requires systematic exclusion of non-immune confounders. Embryonic euploidy should be confirmed, as aneuploid embryos remain the dominant driver of implantation failure (7). The window of implantation must also be considered; displacement detected by transcriptomic assays such as the endometrial receptivity array can mimic immune-mediated failure, although benefits in unselected *in vitro* fertilisation (IVF) populations remain inconsistent (8, 9).

Chronic endometritis and endometrial microbiome imbalance frequently mimic immune dysregulation, confounding interpretation of reproductive immunology testing. CD138-based histology is limited by sampling error and variable diagnostic thresholds (10), whereas molecular assays such as the Endometrial Microbiome Metagenomic Analysis (EMMA) and Analysis of Infectious Chronic Endometritis (ALICE), based on 16S rRNA gene sequencing, provide higher sensitivity and reproducibility for detecting dysbiosis or occult infection (11). Although DNA-based and unable to confirm bacterial viability, these platforms offer essential contextual information. Establishing a molecularly verified, infection-free endometrium should therefore precede evaluation of uterine or systemic immune activation in RIF and RPL (12).

Endometriosis and adenomyosis further compromise endometrial receptivity through inflammatory and stromal pathways, typically characterized by elevated TNF-α signalling and cytokine disruption (13). These effects are often compounded by microbiome imbalance, which can impair implantation independently of immune phenotype and should be addressed before or alongside immunologic evaluation (14).

Only after these prerequisites are excluded, and with synchronised sampling across peripheral and uterine compartments, should immune profiling be undertaken. Successful implantation depends on coordinated phase-specific immune adaptation; RIF and RPL arise when this temporal regulation is disrupted at inflammatory priming, tolerance induction, or vascular remodelling. Current practice lacks a coherent method to relate these immune processes to phenotype and timing.

This review therefore focuses on four elements that provide an interpretable framework: cytokine dynamics, peripheral-uterine immune synchrony, natural killer (NK) cell functional phenotype, and phase-specific timing. These were selected because they link mechanistic biology with measurable clinical parameters and allow immune findings to be interpreted within a defined temporal context. Microbiome status, endocrine factors, systemic autoimmunity, and embryonic competence are considered prerequisite or modifying conditions that must be assessed before immune classification is attempted. Within this scope, the model is presented as hypothesis-generating and intended to guide phenotype-stratified research rather than to function as a validated clinical algorithm.

The following sections outline the methodological approach, review the immune physiology of implantation, examine the current therapeutic landscape, and then present the proposed interpretive framework and its clinical implications.

## 2 Methods

This narrative review synthesised current evidence on immune mechanisms relevant to implantation failure, with particular focus on cytokine dynamics, peripheral blood natural killer (pbNK) and uterine natural killer (uNK) cell activity, and their interpretation within the peri-implantation window. Literature was identified through targeted searches of PubMed, Embase, and Google Scholar from inception to September 2025 using terms related to implantation failure, reproductive immunology, cytokines, NK cells, endometritis, endometriosis, adenomyosis, and immune-directed therapies. Reference lists of key articles were also screened to identify additional relevant studies.

Given the breadth of immune mediators implicated in implantation, emphasis was placed on mechanisms interpretable across biological compartments and defined implantation phases, while other emerging pathways are discussed where contextually relevant. Observational studies, mechanistic investigations, clinical trials, and translational data were included when they informed peri-implantation immune physiology or immune-directed intervention. Owing to substantial heterogeneity in assays, endpoints, timing of assessment, and patient selection, formal meta-analysis was not undertaken. Instead, findings were integrated to identify recurrent mechanistic patterns and to examine contexts in which empirical immunotherapy has underperformed due to inconsistent timing, unstratified populations, or inadequate phenotyping.

This synthesis informed the development of a conceptual diagnostic and interpretive framework that aligns immune assessment with implantation physiology. Termed the Elgheriany Reproductive Immunology Framework (ERIF), the model is proposed as a structured research scaffold to support phenotype-stratified investigation and future clinical trials. The acronym ERIF is used here as a descriptive shorthand for the integrative interpretive model proposed in this review rather than as a formal eponymous designation. It is intended to guide interpretation of immune findings and generate testable hypotheses rather than to function as a prescriptive clinical protocol. This review did not follow a formal systematic review or meta-analytic protocol.

## 3 The peripheral-uterine immune axis in implantation

A clear understanding of the immune pathways governing implantation is essential before considering targeted intervention. Among these pathways, cytokine trajectories represent the most reproducible signals. Physiological implantation involves an initial inflammatory priming followed by a rapid transition toward immune tolerance. This transition is consistently reflected by a rise in interleukin-10 (IL-10) accompanied by suppression of tumour necrosis factor-alpha (TNF-α). In contrast, RIF and miscarriage cohorts demonstrate persistence of TNF-dominant signalling with inadequate IL-10 induction (15).

Dynamic changes in TNF-α and IL-10 over the implantation window therefore carry greater biological relevance than isolated measurements. Assessment of both absolute levels and directional shifts over time better captures failure of immune adaptation. Ratios incorporating IL-10, including TNF-α/IL-10 and IFN-*γ*/IL-10, further enhance discrimination between physiological tolerance acquisition and pathological persistence, although assay heterogeneity and lack of standardized thresholds continue to limit routine clinical translation (16).

Timing and compartmentalization are critical to immune interpretation. IFN-*γ* illustrates this clearly: systemic elevation reflects pathological Th1 activation, whereas locally produced IFN-*γ* from uNK cells is essential for spiral artery remodelling and early placentation (17). Regulatory T cells (Tregs) play a complementary role in enforcing immune tolerance. In murine models, Treg depletion precipitates pregnancy loss, while adoptive transfer restores implantation (18), and human transcriptomic studies demonstrate disrupted Treg-associated signatures in RPL (19). Because immune mediators exert compartment- and phase-specific effects, isolated measurements are insufficient. Meaningful immune interpretation therefore requires synchronized peripheral and endometrial assessment within the implantation window.

NK cells remain the most debated effector population in reproductive immunology. The pbNK cells are predominantly CD56^dim/CD16^+, reflecting a cytotoxic phenotype, whereas uNK cells are CD56^bright/CD16^−, minimally cytotoxic, and essential for spiral artery remodelling, trophoblast invasion, and early placental development (20, 21). This phenotypic and functional divergence underscores the limitation of extrapolating peripheral immune data to the uterine microenvironment.

Misinterpretation has frequently arisen when absolute uNK density, rather than cytotoxic function, was emphasized. Early reports linking “high uNK density” to implantation failure proved poorly reproducible, largely due to inter-laboratory variability and inconsistent quantification (22). Subsequent analyses implicate cytotoxic uNK subpopulations, particularly CD16^+ and CD57^+ subsets, rather than bulk CD56^+ populations, as pathogenic (23). To address these inconsistencies, molecular profiling of endometrial immune transcripts, quantifying CD56, IL-15, IL-18, and TWEAK/Fn-14 expression, has emerged as a reproducible alternative to histologic or flow-cytometric enumeration. This approach stratifies uterine immune activity into low, normal, or high profiles, reflecting deficient, balanced, or overactive immune states with distinct therapeutic implications (24).

In contrast, pbNK activation markers (e.g., CD69) and cytotoxicity assays consistently associate with RIF and RPL (25). Importantly, functional assays permit *ex vivo* assessment of responsiveness to agents such as prednisolone, IVIG, or intralipid, providing a rational means to avoid unnecessary immunosuppression (26). In this context, function, rather than absolute cell number, is the clinically relevant signal.

Implantation physiology integrates these immune mediators into an ordered sequence rather than isolated signals. The initial phase is characterized by inflammatory priming, where cytokines including IL-1β, IL-6, IL-8, and monocyte chemoattractant protein-1 (MCP-1) facilitate blastocyst adhesion, and excessive suppression at this stage risks disrupting a physiological requirement (27). This is followed by a tolerance switch, driven in part by hCG-mediated expansion of Tregs, modulation of regulatory B-cell phenotypes, and epigenetic repression of chemokines such as CXCL10, establishing a Th2 and Treg-dominant milieu (28, 29). In the final stage, angiogenesis and vascular remodelling are sustained by uNK cells, macrophages, and dendritic cells, together with mediators including leukemia inhibitory factor, IL-33/ST2, prokineticin-1, preimplantation factor, and PD-L1 (30–33). These processes are vulnerable to disruption by infection or dysbiosis, where type I interferon signalling can derail immune tolerance and provoke NK hyperactivation (34).

Maternal-fetal genetics introduce a modifying axis that influences local decidual immune activation and trophoblast-uNK interaction. The combination of maternal killer immunoglobulin-like receptor (KIR) AA genotype with fetal HLA-C2 is consistently associated with impaired trophoblast invasion and preeclampsia, whereas activating KIR haplotypes appear protective (35).

Overall, successful implantation depends on coordinated, phase-specific immune transitions across peripheral and uterine compartments rather than isolated immune markers.

## 4 Therapeutic landscape and Its limitations

Over three decades, a wide spectrum of immune-directed therapies has been deployed in RIF and RPL, including corticosteroids, IVIG, intralipid, calcineurin inhibitors, hydroxychloroquine, biologics, cell-based therapies, probiotics, and molecular adjuncts. Despite extensive use, consistent clinical benefit has remained elusive. The central limitation is clear: interventions have rarely been matched to immune phenotype, implantation phase, or biological plausibility. The result has been a succession of negative randomised trials in unstratified populations, in which true responders were diluted by immunologically normal patients (36).

### 4.1 Systemic immunosuppression and its limitations

Corticosteroids remain the most widely prescribed immunomodulatory intervention in RIF and RPL. Their rationale is intuitive: suppression of Th1 cytokines such as TNF-α and IFN-*γ*, together with dampening of NK activity. However, this effect is biologically blunt. While pbNK activity may be reduced, uNK cells are indispensable regulators of spiral artery remodelling, and indiscriminate suppression risks impairing placentation (21, 37).

Small, uncontrolled or feasibility studies have suggested a possible benefit of corticosteroid use in selected populations, particularly among women with elevated uNK cell density (38). More recent evidence evaluating combined immunomodulatory regimens indicates that aspirin plus prednisone or prednisolone may improve clinical pregnancy and implantation rates in IVF and RIF populations, with a potentially enhanced effect in ANA-positive subgroups (39). However, these findings are derived from heterogeneous studies with limited enrollment, and the impact on live-birth outcomes remains uncertain, underscoring the need for adequately powered randomized controlled trials. Although the smaller study reported no significant metabolic adverse signals (38), a large Australian *in vitro* fertilisation/intracytoplasmic sperm injection (IVF/ICSI) cohort linked first-trimester corticosteroid exposure to increased risk of congenital anomalies, including cryptorchidism, hypospadias, and talipes (40). In clinical practice, low-dose prednisolone (10–20 mg/day) initiated shortly before embryo transfer and withdrawn by 8–10 weeks is sometimes used, yet evidence supporting empiric application remains weak (41).

Similar limitations apply to calcineurin inhibitors such as tacrolimus and cyclosporine. Small cohorts with aberrant Th1/Th2 ratios have suggested potential benefit (42, 43), but robust randomised data are lacking, and concerns regarding hypertension, nephrotoxicity, and glucose intolerance restrict use outside research settings. Hydroxychloroquine, which modulates Toll-like receptor signalling, is well established in lupus and antiphospholipid syndrome (44), but has not demonstrated consistent benefit in idiopathic RIF or RPL. In a recent French prospective multicentre registry, hydroxychloroquine use in women with RPL was not associated with improved live-birth rates compared with standard care (45). Collectively, these agents illustrate the hazards of applying systemic immune suppression without phenotype-directed selection.

### 4.2 Biologic and plasma-derived immunotherapies

Biologic and plasma-derived immunotherapies have followed a similar trajectory. IVIG has been proposed to block Fc receptors, expand Tregs, and neutralize autoantibodies (46). Early uncontrolled studies suggested that modulation of abnormal cytokine profiles with IVIG might improve implantation in immunologically selected or secondary recurrent RPL cohorts (47, 48). However, systematic reviews and meta-analyses have not demonstrated a reproducible live-birth benefit in women with primary RPL or in unselected IVF populations lacking immune stratification (49).

Intralipid, promoted as a lower-cost alternative to IVIG, has been shown *in vitro* to reduce NK cytotoxicity and promote Th2-skewed cytokine production (50). Early uncontrolled reports suggested improved outcomes in women with elevated NK activity. However, meta-analytic synthesis has demonstrated no significant effect on miscarriage rates and only suggestive benefit in women with RIF or RPL, pending confirmation in larger trials (51), and a recent meta-analysis confirmed the absence of reproducible benefit across heterogeneous studies (52). The precise *in vivo* mechanism of intralipid therefore remains uncertain.

### 4.3 Cytokine-Targeted biologic therapies

TNF-α inhibitors offer a mechanistically plausible approach to dampening Th1-dominant immune activation. Evidence in reproductive medicine remains preliminary and phenotype-specific; however, retrospective analyses in women with inflammatory reproductive conditions, including endometriosis and adenomyosis, have reported improved implantation and pregnancy rates with peri-implantation adalimumab treatment (53). Additional evidence includes a prospective single-arm study in RIF suggesting a potential implantation benefit (54), and a randomised controlled trial reporting improved outcomes in immune-mediated RPL (55). Despite biological plausibility, these studies are limited by small sample sizes, heterogeneous immunological criteria, and lack of independent replication.

The ESHRE RPL guideline does not address TNF-α inhibitors specifically, but concludes that immunomodulatory therapies, including biologics, lack sufficient evidence for routine use and should be restricted to research settings (56). Certolizumab pegol appears obstetrically safe, with pharmacokinetic data demonstrating minimal placental transfer (57) and registry studies reporting no increase in congenital anomalies (58); however, these safety data are derived from autoimmune disease cohorts rather than fertility populations. Overall, TNF-α blockade remains investigational for RIF and RPL, with insufficient evidence to justify routine clinical use.

### 4.4 Cellular and regenerative immunomodulatory approaches

Cellular therapies have been explored as locally acting immunomodulators. Intrauterine infusion of autologous peripheral blood mononuclear cells (PBMCs) can induce local release of IL-10 and granulocyte-macrophage colony-stimulating factor GM-CSF (59), and systematic reviews and meta-analyses suggest improved pregnancy and live-birth rates in women with RIF (60). However, substantial heterogeneity exists in cell preparation, dose, activation protocols, and timing, limiting reproducibility and clinical standardization.

Mesenchymal stromal cells, derived from bone marrow or endometrium, have shown promise in pilot studies for refractory thin endometrium (61). Platelet-rich plasma delivers a concentrated milieu of cytokines and growth factors, with recent randomised trials and meta-analyses reporting benefit in thin endometrium and RIF (62). Granulocyte colony-stimulating factor has been investigated with similar objectives, promoting angiogenesis and modulating NK and Tregs balance, although systematic reviews highlight inconsistent outcomes (63). Collectively, these approaches remain biologically attractive but should be regarded as experimental pending standardized protocols and robust validation.

### 4.5 Endometrial receptivity, hormonal modulation, and molecular adjuncts

Endometrial receptivity and hormonal modulation remain among the more established areas of intervention. The endometrial receptivity array (ERA) was developed to detect displacement of the window of implantation (WOI), and early studies in RIF cohorts suggested WOI displacement in approximately 25%–30% of patients (64). However, in unselected IVF and frozen embryo transfer populations, randomised trials and meta-analyses have not demonstrated a significant improvement in live-birth rates with ERA-guided personalised embryo transfer (65). Potential clinical utility therefore appears confined to selected phenotypes, such as women with RIF and adenomyosis, in whom WOI displacement has been reported to occur at approximately twice the frequency observed in controls (66).

Progesterone remains indispensable, not only for luteal support but also for its immunological actions mediated in part through progesterone-induced blocking factor (PIBF). Low luteal progesterone levels have been consistently associated with implantation failure (67). Human chorionic gonadotropin (hCG) is increasingly recognised as an immunomodulator beyond luteal support, promoting recruitment of Tregs and B cells, repressing CXCL10 expression, and enhancing IL-10 production (68, 69). Clinical trials and meta-analyses suggest that peri-transfer hCG may improve implantation in selected RIF cohorts, although heterogeneity in dose, route, and timing contributes to inconsistent outcomes (70).

Molecular mediators including leukemia inhibitory factor (71), IL-33/ST2, prokineticin-1, and preimplantation factor (PIF) are mechanistically compelling regulators of implantation and tolerance (72–74), but currently lack validated clinical application.

### 4.6 Infection and the endometrial microbiome

Infection and the endometrial microbiome represent a more actionable domain. Chronic endometritis, frequently associated with organisms such as *Gardnerella or Enterococcus,* is a well-established cause of implantation failure, and confirmation of eradication has been shown to improve reproductive outcomes (75). Dysbiosis of the endometrial microbiota can activate type I interferon pathways and promote NK hyperactivation (76). Correction of these disturbances using antibiotics or targeted probiotic strategies, including *Lactobacillus salivarius,* has shown encouraging preliminary results (77). Addressing infection and microbiome status prior to immunomodulation is therefore essential to avoid diagnostic and therapeutic misattribution.

Taken together, these interventions reveal a recurring paradox. Many approaches are mechanistically compelling and supported by pilot data, yet their apparent benefits often disappear when evaluated in unstratified populations. This pattern closely parallels the experience in oncology prior to the adoption of biomarker-driven treatment paradigms (78, 79). The implication is not that immune therapies lack biological relevance, but that their clinical benefit depends on appropriate patient selection and precise timing. These observations support a shift toward a unified approach in which immunotherapy is considered only when an immune disturbance is demonstrable within the implantation window, and where intervention is aligned with the physiological sequence of implantation. This rationale underpins the development of a phenotype and phase directed framework.

## 5 Phenotype and phase directed framework (ERIF)

The ERIF is proposed as a structured interpretive model to address limitations of empirical immunotherapy by conditioning intervention on demonstrable immune dysfunction within a synchronised implantation window. Before immune profiling is undertaken, non-immune confounders must be excluded. Embryonic competence should be established, either through confirmation of euploidy or through clinical assessment when aneuploidy is not the primary driver of failure (7). Endometrial infection and dysbiosis should be excluded using reliable microbiological or molecular methods, with chronic endometritis assessed concurrently (10–12). The structural and inflammatory effects of adenomyosis and endometriosis should be considered (13); progesterone sufficiency verified (82); and assessment of the window of implantation reserved for cases in which displacement is clinically suspected (66). Systemic autoimmune evaluation is recommended when history or phenotype suggests risk, since unrecognised autoimmune disease may mimic or contribute to reproductive immune disturbance (2, 56). Only after these prerequisites are excluded can immune findings be more reliably interpreted within the framework.

A cycle-matched assessment at progesterone plus five to seven in programmed cycles or LH plus seven to nine in natural cycles captures both systemic and endometrial immunity within the implantation window (27) and allows phenotypic classification into physiological, short, long, ultra-long, or low-activation patterns. Post-transfer monitoring is guided primarily by cytokine dynamics and ratios, which more reliably reflect the transition from inflammatory priming to immune tolerance than isolated measurements (15). The uNK phenotype functions as the principal safety checkpoint: suppression of non-cytotoxic CD56^bright uNK risks impairing implantation, whereas enrichment of cytotoxic CD16^+ or CD57^+ subsets supports more sustained modulation (80). This peri-implantation assessment establishes a working baseline immune phenotype to guide management rather than serving as a definitive diagnostic endpoint. Within ERIF, peripheral cytokine dominance is used to characterise the presence and direction of immune activation, uNK phenotype informs phenotype duration (short vs. long), and systemic autoimmune markers may modify escalation risk toward ultra-long phenotypes. Low-activation states are characterised by blunted cytokine priming and suppressed cytotoxic function.

Therapeutic decisions are guided by immune phenotype and timing, with the degree of immunomodulation calibrated to measurable dysfunction. NK cytotoxicity assays may inform initial treatment selection or escalation (50). Short phenotypes generally warrant only brief peri-implantation modulation, whereas long and ultra-long phenotypes reflect persistent immune activation and may justify continuation of suppression into early gestation until IL-10 rises and TNF-α and IFN-*γ* decline (3). Low-activation states, in contrast, may benefit from immune support rather than suppression. Such phenotypes may reflect inadequate inflammatory priming at implantation. Potential mechanisms include reduced NK functional activity, impaired cytokine induction, altered dendritic or macrophage signalling, or endocrine-immune dysregulation affecting early implantation signalling (24). Interventions are intended to be finite and proportionate, with treatment withdrawal typically considered by eight to ten weeks once immune tolerance is established.

Progesterone optimisation and low-dose hCG act as endocrine-immune synchronisers reinforcing immune tolerance, with hCG functioning as a physiological facilitator of the inflammatory-to-tolerance transition rather than a primary immunotherapeutic intervention (81–83). Its role is adjunctive and phase-specific, introduced in phenotypes with identified immune activation (short, long, and ultra-long) to support tolerance induction once excessive activation has been appropriately stratified, without defining immune phenotype or driving escalation.

Following embryo transfer, immune monitoring within ERIF focuses on cytokine trajectories rather than repeated cytotoxicity testing. Cytokines are reassessed at predefined intervals, typically two-weekly in long and ultra-long phenotypes, to evaluate directional immune adaptation. When immune markers improve but are not fully normalised, cautious continuation or dose reduction is favoured over escalation, unless there is clear evidence of persistent cytotoxic activation. This approach balances biological plausibility, maternal-fetal safety, assay variability, and clinical feasibility, and avoids prolonged empiric immunosuppression in the absence of demonstrable ongoing harm.

**Figure 1:**
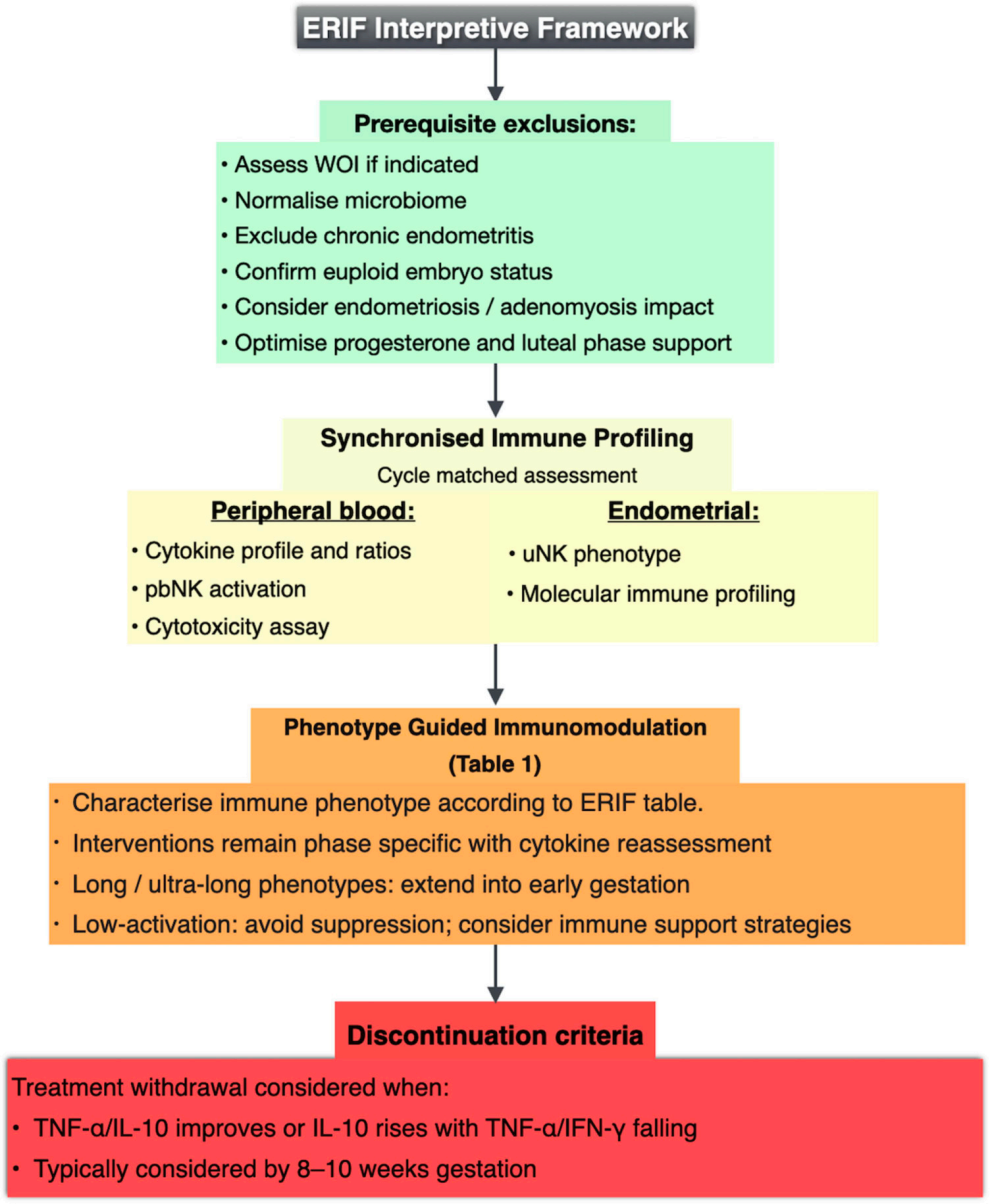
ERIF interpretive framework illustrating prerequisite exclusion, cycle-matched immune assessment, phenotype characterisation, and phase-guided immunomodulation with defined reassessment and discontinuation considerations. Phenotypes are characterised by immune patterns over time rather than by single cytokine values, fixed cut-off thresholds, or uniform NK gating assumptions.

This structure outlines a phenotype-directed interpretive pathway aligning immune mechanisms with implantation timing. Figure 1 illustrates the ERIF framework, and Table 1 summarises phenotype characteristics, aligned interventions, and discontinuation considerations.

**Table 1:** ERIF phenotypes with aligned immune characteristics, phase guided interventions, and discontinuation considerations.

Phenotype	Composite findings	Initial action	Adjuvants/Activation	Discontinuation criteria
Physiological	TNF-α/IL-10 normal; No pbNK activation; Non-cytotoxic uNK; Normal microbiome; Autoimmune screen negative.	No immunotherapy	High-range Progesterone	No immunetherapy
Borderline activation (Short)	Mild TNF-α/IL-10 tilt; Mild pbNK activation (CD69↑); Mild elevation in cytotoxicity assay; Intact uNK phenotype. Autoimmune screen negative.	Prednisolone 5–15 mg, pre-ET (day −7 to −3), cytotoxicity-guided; taper 3–5 days after ET.	Optional intralipid; hCG boluses; Progesterone optimisation.	Re-check cytokine profile at ET+ 5–7. Stop once IL-10 increases and TNF-α decreases, or when the TNF-α/IL-10 ratio normalises.
Sustained Th1 dominance (Long)	Moderate TNF-α/IL-10 dominance; Moderate pbNK activation + cytotoxicity assay; Cytotoxic uNK phenotype; ANA negative or weak positive; Reflex ENA negative.	Prednisolone 10–20 mg pre-ET (day −7 to −5), with optional IVIG or intralipid. Tacrolimus pre-ET (day −3 to −1) when cytotoxic uNK is present or contributes significantly to the phenotype.	hCG boluses; Progesterone optimisation.	Monitor TNF-α/IL-10 every 10–14 days until 8–10 weeks Taper once IL-10 increases and TNF-α decreases, or when the TNF-α/IL-10 ratio normalises.
Multi-axis Hyperactivation (Ultra-long/Refractory)	High TNF-α/IL-10 dominance; High pbNK activation + elevated cytotoxicity assay; Cytotoxic uNK phenotype; ANA positive and/or reflex ENA positive.	Immune normalization using minimal axes: IVIG or anti-TNF → monitor q14 days; defer FET ×2 weeks after stability. Add prednisolone only if Th1 tilt persists. Tacrolimus pre-ET (day −3 to −1) for cytotoxic uNK ± Th1 tilt (replaces steroids).	hCG boluses; Progesterone optimisation.	Monitor TNF-α/IL-10 every 10–14 days until 8–10 weeks Taper once IL-10 increases and TNF-α decreases, or when the TNF-α/IL-10 ratio normalises.
Low-activation	Low TNF-α/IL-10; Oversuppression in cytotoxicity assay; Low uNK activity; Blunted cytokine priming.	No immunotherapy	Endometrial scratch; PRP; pre-ET (day −7 to −5) GM-CSF; pre-ET (day −3 to 0) UPSI around ET to restore priming.	No immunotherapy

Overview of phenotype classification integrating cytokine dynamics, NK functional status, autoimmune context, and endometrial findings to align phase-guided interventions with monitored withdrawal. Phenotype-intervention associations are conceptual and hypothesis-generating and require prospective validation in phenotype-stratified clinical studies.

## 6 Discussion

Reproductive immunology remains characterised by strong mechanistic insight but inconsistent clinical translation. This manuscript proposes a conceptual interpretive framework to support phenotype- and phase-stratified investigation. Its purpose is to organise existing evidence into a structured model that may guide future mechanistic studies and randomised trials, rather than to introduce a validated therapeutic algorithm.

Recent multicentre data reaffirm that immune abnormalities are frequently detected in women with recurrent reproductive failure, yet evidence from 2025 cohort analyses shows that findings derived from retrospective and empiric designs cannot reliably predict treatment response (84). The apparent success of broad immunotherapy “cocktails,” typically combining aspirin, heparin, corticosteroids, or IVIG, may reflect referral bias and multimodal empiricism rather than reproducible efficacy. This polarisation has led some to dismiss immune modulation entirely while others persist with broad suppression without mechanistic justification. Both positions overlook that immune dysregulation is clinically relevant for a defined subset of patients, but translation demands phenotype and phase specific precision rather than empiric generalisation.

Mechanistic evidence now provides a consistent rationale for stratification. Cytokine trajectories, particularly TNF-α/IL-10 and IFN-γ/IL-10 ratios, are reproducible markers of tolerance induction, yet miscarriage cohorts remain locked in a TNF-dominant inflammatory state at implantation (4). Peripheral NK activation markers such as CD69 and cytotoxicity assays correlate with recurrent loss, but predictive value improves only when interpreted alongside uNK phenotypes in which cytotoxic CD16 and CD57 subsets, not bulk CD56 populations, appear pathogenic (20–23). Implantation physiology further demonstrates that an early inflammatory burst is required for apposition, while persistence without transition to tolerance predicts failure (15, 18, 27). These observations may help explain why unstratified RCTs of prednisolone or IVIG have frequently returned null results, as potential responders may be diluted within immunologically heterogeneous populations (84).

Comparable lessons have emerged in transplantation, where durable tolerance required balanced regulatory and effector control rather than blanket suppression (78); in autoimmunity, where disease-modifying therapies benefit only phenotypically defined subsets (85); and in oncology, where checkpoint inhibitors transformed outcomes only within biomarker-selected contexts (79). Reproductive immunology may be approaching a similar inflection point.

The economic and clinical implications reinforce this shift. IVIG carries substantial cost and procedural risk without consistent benefit, and intralipid remains mechanistically uncertain with no reproducible live-birth advantage (5, 86). Prolonged corticosteroid exposure increases gestational diabetes and hypertensive morbidity, underscoring the risks of prolonged empiric suppression (40, 87). In this context, a composite panel integrating cytokine ratios, pbNK activation, and targeted uNK phenotyping may offer a more structured and potentially cost-conscious approach to stratification (88). In practice, implementation would depend on access to standardised laboratory platforms and local expertise, and feasibility may vary between centres. Serial cytokine monitoring may represent a comparatively accessible component of this approach, as it is relatively inexpensive and can be repeated longitudinally to assess directional immune adaptation.

Future progress depends on second-generation RCTs that prospectively stratify by immune phenotype, harmonise assays across laboratories, and include mechanistic endpoints such as cytokine trajectories and NK functional shifts alongside live birth. Multi-omics approaches, including transcriptomics, microbiome profiling, and epigenetic repression signatures, may refine classification further (89–91). Artificial intelligence may eventually integrate these datasets into predictive models, but until then, composite immune panels may represent a pragmatic entry point under current technical and financial constraints.

Emerging mediators highlight additional opportunities for future investigation. IL-33/ST2 (72), prokineticin-1 (73), PIF (74), and PD-L1 (92) regulate receptivity and immune tolerance (93, 94) but currently remain predominantly mechanistic or preclinical targets rather than clinically actionable interventions. Immune checkpoints are similarly promising: reduced Tim-3 expression on decidual macrophages and T cells has been associated with RPL (95, 96), while TIGIT signalling promotes IL-10 production and Th2 polarisation. Although TIGIT-Fc fusion proteins show experimental promise in preclinical models (97), translation into reproductive medicine will require standardised assays, phenotype-stratified trial design, and demonstration of safety and reproducibility before clinical consideration.

Although formal validation is essential, early clinical observations suggest that this framework is operationally feasible. In observational settings, patients with cytokine imbalance but normal NK activation have been managed using short, low-dose prednisolone with hCG support, whereas those with suppressed cytotoxicity assays did not appear to benefit from non-stratified immune suppression. In selected cases, withholding immunomodulation until biochemical pregnancy and applying minimal, time-limited intervention in response to rising cytokines was used to support immune tolerance while limiting unnecessary exposure. These observations are exploratory, hypothesis-generating, and not generalisable, but they illustrate feasibility and underscore the need for prospective, phenotype-stratified evaluation.

Recent molecular immune-profiling RCT data are consistent with this trajectory: in a randomised trial involving a large IVF cohort, phenotype-directed endometrial immune profiling was associated with higher live-birth rates than empiric care. However, as these findings derive from a single study using a specific analytical platform, external replication and validation across centres remain necessary before broad clinical adoption (24).

It is important to acknowledge that the predictive value and clinical benefit of endometrial receptivity array (ERA), Endometrial Microbiome Metagenomic Analysis (EMMA), and Analysis of Infectious Chronic Endometritis (ALICE) remain debated. While these tools may provide contextual information in selected cases, current evidence does not support their routine application in unselected populations, and their clinical utility varies depending on patient phenotype and study design. Similarly, when embryonic competence is considered within this framework, euploidy assessment ideally refers to validated preimplantation genetic testing methodologies; however, variability in platform accuracy, mosaicism interpretation, and clinical thresholds continues to generate ongoing debate. Accordingly, ERIF does not depend on any single diagnostic platform but emphasises cautious interpretation within the broader clinical context.

A key strength of this review is the integration of mechanistic, clinical, and translational evidence into a coherent framework that links immune phenotypes to implantation physiology. Rather than treating cytokines, NK subsets, uterine signalling, and endocrine cues as isolated observations, ERIF synthesises these elements into a timing-based model that can be prospectively tested. By defining clear prerequisites, phenotypic categories, and finite stop rules, the framework offers a structured template that future mechanistic studies and randomised trials can evaluate and refine. Accordingly, ERIF is intended primarily as a research and trial-stratification framework to guide immune interpretation and hypothesis generation, rather than as a prescriptive clinical treatment algorithm. In doing so, it provides a structured basis for moving beyond descriptive immune markers toward evaluable, phase-specific investigation.

This review has several limitations. The proposed framework extrapolates partly from mechanistic and translational studies rather than prospective validation. Inter-laboratory variability, particularly in cytokine assays, cytotoxicity testing, and endometrial immunophenotyping, remains a major barrier to reproducibility (98). Accordingly, ERIF does not propose universal numeric cytokine cut-off values but instead emphasises longitudinal immune trajectories within the same laboratory platform. Biopsy-dependent profiling introduces procedural variability and may influence subsequent immune readouts (99). The absence of standardised endpoints across immunotherapy trials further limits data synthesis and meta-analysis (100).

These constraints underscore the need for harmonised laboratory platforms and validated non-invasive biomarkers. As demonstrated by the evolution of preimplantation genetic testing for aneuploidy, assay calibration and external quality assurance are essential for reliable clinical translation (101). Accordingly, ERIF emphasises longitudinal interpretation within the same laboratory, focusing on directional cytokine patterns rather than absolute thresholds that vary across platforms.

Given the breadth and heterogeneity of reproductive immunology research, integration of all immune mediators within a single interpretive model is neither feasible nor clinically actionable. Selectivity was therefore necessary to preserve structural coherence. As a result, the framework does not comprehensively evaluate all emerging immune pathways or biomarker platforms and may not capture rare or context-specific mechanisms of implantation failure. Similarly, earlier immunological influences such as paternal antigen exposure or seminal plasma signalling fall outside the peri-implantation focus of ERIF and are therefore not incorporated into the operational framework.

## 7 Conclusion

The principal contribution of this work is the introduction of an integrated interpretive framework designed to clarify when immune findings are clinically meaningful, which phenotypes may warrant intervention, and when treatment withdrawal should be considered. Progress in reproductive immunology will require moving beyond blanket immune suppression and guideline skepticism toward phenotype- and phase-directed immunomodulation, structured around demonstrable dysfunction, initiated within the implantation window, and reassessed as immune tolerance develops. Continued advancement will require standardised laboratory practice, global collaboration, and rigorously stratified clinical trials. Until then, clinicians should proceed cautiously while recognising that one-size-fits-all immune suppression is unlikely to address phenotype- and phase-specific dysfunction.
